# Chiropractic pediatric patient management and interdisciplinary collaboration: a descriptive cross-sectional study of chiropractors in Quebec

**DOI:** 10.1186/s12998-022-00464-y

**Published:** 2022-12-13

**Authors:** Chantale Doucet, Élisa Dubuc, Camille Imbeau, Katherine A. Pohlman, Marc-André Blanchette

**Affiliations:** 1 Département de Chiropratique, Université du Québec à Trois-Rivières (UQTR), 3351, Boul. Des Forges, C.P. 500, Trois-Rivières, QC G9A 5H7 Canada; 2 Center, Parker University, Dallas, TX USA

**Keywords:** Children, Pediatric, Chiropractic, Interdisciplinary, Practice, Survey

## Abstract

**Background:**

Worldwide, many patients, including minors, seek chiropractic care. The purpose of this study was to investigate the practice characteristics of chiropractors who treat pediatric patients in Quebec, Canada.

**Methods:**

We conducted a web-based cross-sectional survey of all licensed chiropractors working in Quebec (Canada). Data were collected using an adapted questionnaire. Descriptive statistics were produced for all the variables collected.

**Results:**

Among our 245 participants (response rate: 21%), 63% were women, and half defined themselves as general musculoskeletal (MSK) health care practitioners. Nearly all participants reported seeing 0–5 new pediatric patients/week, and the most common pediatric age group was 6–12 years old (57%). Pediatric patients were most commonly referred by family members and “word of mouth”. The respondents most frequently indicated that they strongly agreed with statements affirming their confidence in their own diagnostic capacities regarding MSK disorders with respect to all age groups as well as non-MSK disorders with regard to young teens. They reported a moderate level of agreement with similar statements concerning the diagnosis of non-MSK disorders in newborns, preschoolers, and children. Chiropractors rarely referred their pediatric patients to a nurse/family doctor or a pediatrician. When presented with potential pediatric red flags, the respondents commonly indicated that they would refer the patient to a physician in an emergency situation or for comanagement.

**Conclusion:**

Chiropractors in Quebec are confident in their diagnoses of pediatric MSK conditions and refer patients to physicians in the rare event of a worrisome presentation. However, some chiropractors may have expectations that are unsupported by evidence regarding the diagnosis and management of non-MSK complaints.

**Supplementary Information:**

The online version contains supplementary material available at 10.1186/s12998-022-00464-y.

## Background

Patients worldwide seek chiropractic care, including children under the age of 18 years old [1–4]. Recent studies have reported that the rates at which the pediatric population (≤ 18 years) and the older adult population (≥ 55 years) seek chiropractic services are lower than that of the general adult population [5]. Nonetheless, the estimated global prevalence of the utilization of chiropractic care at the age of 12 months is 8.1% (interquartile range [IQR]: 3.8–20.00), with a lifetime use of 11.1% (IQR: 4.0–21.6) for children [6]. The most common reasons for pursuing chiropractic care are musculoskeletal conditions, including back pain and headaches [6, 7]. A prevalence of 55.5% (95% confidence interval [CI] 52.1–59.0%) was estimated by a Danish study of adolescents (8–15 years old) suffering from back pain [7]. However, persistent crying (median: 19.8%, IQR: 10.0–29.6); gastrointestinal conditions (mean: 17.5%, IQR: 10.7–40.3); ear, nose, and throat conditions (mean 8.3%, IQR: 3.0–10.0); and asthma (mean: 5.3%, IQR: 2.0–8.5) are clinical complaints that are mostly observed in the youngest group of patients (less than 2 years old) [6, 8–12]. Specifically, in Canada, Mior et al. [4] reported that 5.5% of the patients encountered at chiropractors’ offices in Ontario in 2014 were below 15 years of age.

Despite these data, the provision of chiropractic care to children is controversial, and a position statement was issued by the Canadian Pediatric Society in 2002 to warn about the effectiveness and safety of such care [13]. Twenty years have passed since the publication of this Canadian statement; over that time, the chiropractic profession has evolved. Recent studies investigating the attitudes of Canadian family physicians toward chiropractic care have highlighted their positive opinions regarding musculoskeletal conditions over the past decade [14–16]. According to these results, medical doctors continue to exhibit concerns regarding the possibility of referring pediatric patients to chiropractic services in general. With the intention of informing both the medical and chiropractic professions of the behavior of chiropractors in Quebec regarding pediatric patients, this study became imperative.

Conducting a cross-sectional survey on chiropractic care in Quebec can inform current pediatric chiropractic practice trends and patterns and help promote a better understanding of the profession. This information provides a means of advancing interprofessional relationships between chiropractors and pediatric health care providers. The purpose of this study was to document the practice profile of chiropractors in Quebec who treat children below 18 years of age. More specifically, we aimed to describe the (1) demographic characteristics of chiropractors who treat children in Quebec; (2) patients’ characteristics by age group; (3) knowledge of red flags and referral patterns; (4) pediatric educational training; (5) level of certainty in the chiropractor’s clinical impressions; (6) the pediatric conditions treated; and (7) the nature of interdisciplinary collaboration between chiropractors and other primary care and allied professions.

## Methods

### Study design

We conducted a web-based cross-sectional survey of all licensed chiropractors working in Quebec (Canada).

### Survey instrument

The questionnaire developed by Siegenthaler [17] was tested for content validity prior to its use in a cross-sectional study aimed at describing pediatric practice in the context of a Swiss chiropractic clinic. With respect to the current study, this extensive questionnaire seemed to us to serve as a good starting point because it largely suits our research objectives. However, adaptations were deemed to be necessary to understand chiropractic practice in Quebec. The Siegenthaler questionnaire [17] was first translated into the French-Canadian language (with author permission) by a chiropractic intern (CI) and adapted by the research team (CD, CI) with the assistance of an epidemiologist and content experts. Following the recommendation of Epstein et al. [18], no back translation was performed due to its lack of added value compared with the sole use of expert committee review. The questionnaire was enhanced by adding concepts from previous studies in order to better reflect pediatric chiropractic practice in Quebec [5, 7–13, 19, 20]. We added a section concerning the management of common pediatric red flags for chiropractic care, which were curated within a best practices document [21].

The final questionnaire included a consent form and 47 items focusing on eight themes (Additional file 1):

- Demographic data (4 items)
- Practical characteristics (5 items)
- Collaborative practices (4 items) and referral patterns (5 items)
- Diagnostics and recommendations (2 items)
- Patient characteristics (20 items)
- Management of red flags (3 items)
- Professional affiliation and continuing education (3 items)
- Future avenues for research regarding the pediatric population (1 item).

The results for the last two themes will be reported in a subsequent publication.

### Pilot testing

Our preliminary questionnaire was pilot tested based on a convenience sample of 29 chiropractors (May–June 2019) who were recruited on social media via two Facebook pages targeting chiropractors with pediatric interests in Quebec [22]. Respondents were invited to provide any type of feedback that they deemed to be appropriate for improving the clarity and exhaustivity of the questionnaire. Face validity was assessed, and minor improvements were made to the preliminary questionnaire based on the feedback provided by the respondents [22].

### Participants and administration

A web-based cross-sectional survey was sent to all members of the “Ordre des Chiropraticiens du Québec” (OCQ). In the province of Quebec, the mandate of the OCQ is to ensure public protection and excellence in chiropractic practice. Membership in the OCQ is a requirement to practice chiropractic within the province. To obtain an adequate portrait of Quebec’s chiropractic pediatric care and enhance the feasibility of the study, new graduates (those with less than one year of experience), practitioners without pediatric patients, chiropractors practicing outside of Quebec, or individuals without public contact information were excluded. The ethics board for research involving humans of the Université du Québec à Trois-Rivières (UQTR) deemed that an ethical certification was not warranted due to the confidential nature of the data collection process. The individuals involved were not considered to be participants for the purposes of the Tri-Council Policy Statement: Ethical Conduct for Research Involving Humans—TCPS 2 (2018), article 2.1.

### Data collection

In July 2019, an email invitation was sent to all members of the OCQ with publicly available contact information asking them to complete our survey on the SurveyMonkey platform (1999–2021 SurveyMonkey©). Participants were also solicited on social media via two Facebook pages targeting chiropractors with pediatric interests in Quebec [23]. Participation was voluntary and confidential. Up to three email remainders were sent to nonresponders. Given the lower-than-expected response rate, given that data collection took place during the first year of the COVID-19 pandemic, we initiated a second round of data collection on May 17, 2021 [24, 25]. The list of OCQ members was updated before we resumed the second round of data collection. Again, three weekly email reminders were sent to nonresponders. Partial responders were contacted by phone to supply missing information [23]. The questionnaire was completely closed on June 5, 2021.

### Data analysis

Descriptive statistics (frequencies and percentages) were generated for all items included in the questionnaire. Statistical analysis was conducted using SPSS statistical software, version 27 (IBM SPSS Inc. Armonk, New York).

## Results

**Fig. 1:**
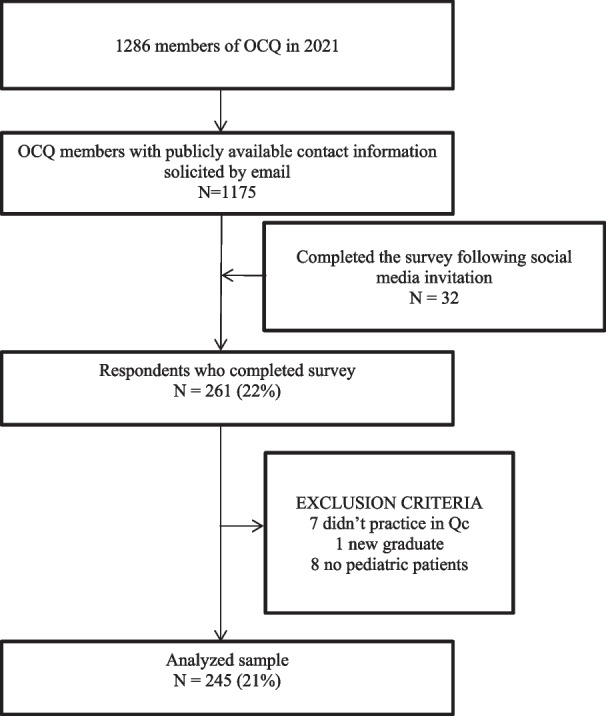
Flowchart of the members of the Quebec’s College of Chiropractors (OCQ) included in the study

Among the 1175 members of the OCQ who were invited to participate in 2021, 261 participants completed our survey. The average completion time was 30 min. Sixteen participants did not meet the inclusion criteria. The final response rate was 21% (n = 245 OCQ respondents) (Fig. 1). The demographic data, practice characteristics, and collaborative practices of respondents are presented in Table 1. When compared to the available statistics for all licensed chiropractors in Quebec [20], our sample included more women (63% vs. 46%) and individuals with the same number of years of experience (mean: 16 years vs. 16 years).

**Table 1:** Demographics, practice characteristics, and collaborative practices (N = 245)

	N	%
**Demographics**		
Sex		
Women	154	62.9
Men	91	37.1
Years in practice (mean = 15.7, SD = 11.3)		
[0–5]	55	22.4
[6–10]	39	15.9
[11–15]	43	17.6
[16–20]	43	17.6
[21–25]	16	6.5
[26+]	49	20
**Practice characteristics**		
Average number of pediatric consultations per week		
[0–5]	112	45.7
[6–10]	63	25.7
[11–15]	36	14.7
[16–20]	21	8.6
[21 +]	13	5.3
Average number of new pediatric patients per week		
[0–5]	243	99.2
[6–10]	2	0.8
Age groups most frequently treated (2 answers required)		
[0–6 months]	85	34.7
[7–23 months]	68	27.8
[2–5 years]	40	16.3
[6–12 years]	140	57.1
[13–17 years]	133	54.3
Types of practice		
Sole practitioner	94	38.4
Group of chiropractors	108	44.1
Multidisciplinary with MD	7	2.9
Multidisciplinary without MD	33	13.5
Other	3	1.2
Main objective of chiropractic treatment		
Improve function	116	47.3
Reduce pain	29	11.8
Prevention	18	7.3
Remove subluxations	25	10.2
Improve lifestyle	1	0.4
Improve quality of life	41	16.7
Other	15	6.1
**Collaborative practices**		
Sources of referrals to the chiropractor		
Pediatrician	16	6.5
Pediatric hospital	4	1.6
Physiotherapist	25	10.2
Speech therapist	9	3.7
Occupational therapist	9	3.7
Psychologist	6	2.4
Family doctor	78	31.8
Medical specialist	6	2.4
Another chiropractor	68	27.8
Nurse	67	27.3
Midwife	43	17.6
Nutritionist	7	2.9
Naturopath	11	4.5
Parent/sibling/other family member	232	94.7*
Acquaintance/not family member	222	90.6*
Publicity	83	33.9
Most common group age referred by medical doctors (2 answers required)		
[0–6 months]	90	36.7
[7–23 months]	67	27.3
[2–5 years]	32	13.1
[6–12 years]	87	35.5
[13–17 years]	100	40.8
Frequency of referral to family physician or nurse practitioner		
Never	28	11.4
Rarely (< 1/month)	145	59.2
A few times (1–3/month)	51	20.8
Often (1–2/months)	9	3.7
Frequently (> 2/week)	0	0
Does not apply	12	4.9
Frequency of referral to pediatrician or another medical specialist		
Never	45	18.4
Rarely (< 1/month)	147	60
A few times (1–3/month)	39	15.9
Often (1–2/months)	2	0.8
Frequently (> 2/week)	0	0
Does not apply	12	4.9
Submission of a written report to patient’s pediatrician		
Never	69	28.2
Rarely (< 1/month)	139	56.7
A few times (1–3/month)	21	8.6
Often (1–2/week)	4	1.6
Frequently (> 2/week)	3	1.2
Does not apply	9	3.7
Knowledge transfer activity to mainstream providers regarding pediatric care		
Never	111	45.3
Rarely (< 1–2 times)	80	32.7
A few times (1/2—3 years)	27	11
Often (1/year)	11	4.5
Frequently (> 1/year)	14	57
Does not apply	2	0.8
Knowledge transfer activity to CAM providers regarding pediatric care		
Never	115	46.9
Rarely (< 1–2 times)	64	26.1
A few times (1/2—3 years)	37	15.1
Often (1/year)	12	4.9
Frequently (> 1/year)	15	6.1
Does not apply	2	0.8
Invitation to present about chiropractic pediatric care		
Never	207	84.5
Rarely (< 1–2 times)	22	9
A few times (1/2—3 years)	7	2.9
Often (1/year)	5	2
Frequently (> 1/year)	2	0.8
Does not apply	2	0.8

*CAM* complementary and alternative medicine, *MD* medical doctor, *SD* standard deviation

*Included in the two most frequent sources of referral for 85% of the respondents

Regarding practice characteristics, 45.7% of the participants reported that pediatric patients visited the clinics 0–5 times per week. A total of 99.2% of participants reported seeing 0–5 new pediatric patients per week in their clinics, and the most common pediatric age group was the 6–12-year-old group (57.1%), followed by 13–17-year-old group (54.3%) and the 0–6-month-old group (34.7%). Group practice (44.1%) was the most frequent type of practice. When asked to identify the main objective of their chiropractic treatment, 47.3% of participants indicated that the main objective was to improve function. A total of 24.9% of participants held a “*Diplomate in clinical chiropractic pediatrics*” (DICCP) certification, indicating postgraduate training after passing their board examination; furthermore, 54% of participants were active members of the Quebec Chiropractic Association in Pediatrics and Perinatal Care (AQCPP), and 50.4% defined themselves as general practitioners.

With regard to patterns of referral to the participating chiropractors and their sources of referral, referrals from “parents, siblings or other family members” and by “word of mouth/nonfamily members” were most common. In contrast, referrals from pediatric hospitals, psychologists, medical specialists, and other chiropractors were less frequent (Table 1). Furthermore, the most common ages for referrals to the clinic were 13–17 years old, followed closely by 0–6 months old. The least common age for referrals was 2–5 years old. The frequency of chiropractic referrals to a nurse/family doctor or a pediatrician was reported as rare (< 1/month) in both cases. The frequency of written communication with other health care professionals was reported as rare (< 1/month).

The conditions treated by Quebec chiropractors by age group are presented in Table 2, and chiropractors’ level of certainty concerning pediatric diagnosis are presented in Table 3. Our respondents most frequently indicated “strongly agree” when surveyed regarding their confidence in their diagnosis of MSK disorders in newborns, preschoolers, children and young teens (high school). However, their most frequent answer to the level of certainty concerning the diagnosis of non-MSK conditions was reported as being “neither agree nor disagree” for all age groups with the exception of non-MSK disorders among young teens (high school).

**Table 2:** Percentage of chiropractors reporting treating various conditions for each age group

0–6 months	7–23 months
Torticollis (84%) Use of instruments at delivery (40%) Asymmetry of the head/Plagiocephaly/Brachycephaly (68%) Abnormal movements (66%) Colic/Excessive crying/Irritability (71%) Digestive problems/Stomach pain/Gastroesophageal reflux (69%) Breastfeeding problems (53%) Sleeping problems (44%) Earache/Otitis (70%) Hip dysplasia (15%) Preventive exam (40%) Allergy to bovine proteins (10%) Bronchiolitis (13%) Spastic baby (22%) Paralysis of brachial plexus (14%) Troubles of the jaw (43%) Jaundice (2%) Fever (13%) Troubles of motor development (38%)	Asymmetric/Torticollis (55%) Asymmetry of the head/Plagiocephaly/Brachycephaly (41%) Abnormal movement (52%) Colic/excessive crying/Irritability (29%) Digestive problems/Stomach pain/Gastroesophageal reflux (42%) Sleeping problems (36%) Earache/Otitis (70%) Allergy (6%) Gait problems (57%) Preventive exam (38%) Asthma (16%) Bronchitis (9%) Serous otitis (29%) Autism (5%) Behavioral disorder (7%)

2–5 years	6–12 years
Asymmetric/Torticollis (unresolved) (23%) Asymmetry of the head/Plagiocephaly/Brachycephaly (10%) Motor development (45%) MSK complaint/Pain to the cervical spine (75%) MSK complaint/Pain to the thoracic spine (63%) MSK complaint/Pain to the lumbar spine and pelvis (71%) MSK complaint/Pain to the upper limbs (57%) MSK complaint/Pain to the lower limbs (68%) Posture (62%) Headache/Migraine (44%) Falls (70%) Growth pain (6%) Earache/Otitis (64%) Asthma/Allergy (19%) Preventive exam (47%) Fever (24%) Serous otitis (24%) Bronchitis (8%) Pneumonia (4%) Scoliosis (29%) Bed wetting (36%)	Motor development (27%) MSK complaint/Pain to the cervical spine (86%) MSK complaint/Pain to the thoracic spine (77%) MSK complaint/Pain to the lumbar spine and pelvis (84%) MSK complaint/Pain to the upper limbs (71%) MSK complaint/Pain to the lower limbs (76%) Posture (77%) Scoliosis (66%) Migraines (59%) Trauma injuries (70%) Sports injuries (81%) Growth pain (62%) Earache/Serous otitis (36%) Asthma/Allergies (15%) Concentration/Hyperactivity (27%) Sleeping disorders (24%) Preventive exam (51%) Psychological profile (5%)

13–17 years	
MSK complaint/Pain to the cervical spine (89%) MSK complaint/Pain to the thoracic spine (85%) MSK complaint/Pain to the lumbar spine and pelvis (88%) MSK complaint/Extremities (79%) Posture (80%) Scheuermann disease (20%) Scoliosis (68%) Headache (82%) Dizziness (23%) Sports injuries (87%) Traumatic injury (76%) Concentration/Hyperactivity (23%) Asthma/Allergies (14%) Menstruation pain (30%) Sleeping disorders (23%) Preventive exam (50%) Psychological disorders (7%) Behavioral disorder (9%)	

*MSK* musculoskeletal

**Table 3:** Confidence regarding diagnostic categories and frequent recommendations [N = 245]; N (%)

	Strongly disagree	Disagree	Neither agree nor disagree	Agree	Strongly agree	Missing
MSK disorders in newborns	5 (2.0)	8 (3.3)	40 (16.3)	74 (30.2)	**116 (47.3)**	2 (0.8)
MSK disorders in preschoolers	2 (0.8)	4 (1.6)	11 (4.5)	86 (35.1)	**140 (57.1)**	2 (0.8)
MSK disorders in children (elementary school)	1 (0.4)	0 (0.0)	8 (3.3)	69 (28.2)	**165 (67.3)**	2 (0.8)
MSK disorders in young teens (high school)	0 (0.0)	0 (0.0)	2 (0.8)	57 (23.3)	**184 (75.1)**	2 (0.8)
Non-MSK disorders in newborns	20 (8.2)	49 (20.0)	**77 (31.4)**	53 (21.6)	43 (17.6)	3 (1.2)
Non-MSK disorders in preschoolers	17 (6.9)	41 (16.7)	**80 (32.7)**	62 (25.3)	42 (17.1)	3 (1.2)
Non-MSK disorders in children (elementary school)	14 (5.7)	32 (13.1)	**78 (31.8)**	77 (31.4)	42 (17.1)	2 (0.8)
Non-MSK disorders in young teens	13 (5.3)	28 (11.4)	72 (29.4)	**79 (32.2)**	50 (20.4)	3 (1.2)

*MSK*  musculoskeletal. Bold = mode

Descriptive statistics regarding the screening/management of red flags are presented in Table 4. The six most common presentations in pediatric patients that would prompt a chiropractor to make an immediate referral to a medical doctor or hospital were as follows:

1. Bulging or sulking fontanelles, the inability to wake up a baby, and persistent abdominal pain or abnormal breathing in a child with diabetes.
2. Fever, pain in chest, impaired mental state or other neurological findings.
3. Fracture or dislocation.
4. Signs of dehydration and/or a decrease of 50% of fluid intake over a 24-hour period in a baby or child, persistent vomiting, weight loss of more than 5%.
5. Cold or white lower limbs and/or cyanosis of the lips, febrile petechiae or purpuric rash.
6. Suicidal ideation, neoplasms or noncircumscribed lesions.

**Table 4:** Management of selected signs and symptoms [N = 245]; N (%)

	Immediate referral to hospital	Exclusive chiropractic care	Comanagement	Prefer not to answer	Missing
Absence of primitive reflexes	95 (38.8)	9 (3.7)	**123 (50.2)**	17 (6.9)	1 (0.4)
Weight loss of more than 5%	**123 (50.2)**	4 (1.6)	105 (42.9)	12 (4.9)	1 (0.4)
Impaired mental state, dehydration, abdominal pain, or abnormal breath in a child with diabetes	**232 (94.7)**	1 (0.4)	10 (4.1)	1 (0.4)	1 (0.4)
Ataxia	**164 (66.9)**	3 (1.2)	72 (29.4)	5 (2.0)	1 (0.4)
Vomiting bile	**200 (81.6)**	4 (1.6)	30 (12.2)	9 (3.7)	2 (0.8)
Bulging or sulking fontanelle	**189 (77.1)**	7 (2.9)	39 (15.9)	7 (2.9)	3 (1.2)
Fracture or dislocation	**224 (91.4)**	0 (0.0)	19 (7.8)	1 (0.4)	1 (0.4)
Chest discomfort of low to medium intensity	**105 (42.9)**	21 (8.6)	**105 (42.9)**	12 (4.9)	2 (0.8)
Cold or white lower limbs and/or cyanosis of the lips	**222 (90.6)**	1 (0.4)	15 (6.1)	5 (2.0)	2 (0.8)
Convulsions with no prior history or association with cranial traumas	**216 (88.2)**	2 (0.8)	25 (10.2)	1 (0.4)	1 (0.4)
Dizziness	53 (21.6)	28 (11.4)	**160 (65.3)**	3 (1.2)	1 (0.4)
Dyspnea combined with nasal flaring or significant increase of breathing rate	**206 (84.1)**	2 (0.8)	28 (11.4)	8 (3.3)	1 (0.4)
Blood in stool	**185 (75.5)**	0 (0.0)	55 (22.4)	2 (0.8)	3 (1.2)
Fever higher than 38° C (rectal) in a child of more than 90 days of age	85 (34.7)	20 (8.2)	**125 (51.0)**	11 (4.5)	4 (1.6)
Fever, pain in chest, impaired mental state, or other neurological findings	**228 (93.1)**	1 (0.4)	13 (5.3)	1 (0.4)	2 (0.8)
Fever equal or above 40° C, particularly associated with a rapid increase	**216 (88.2)**	3 (1.2)	24 (9.8)	243 (99.2)	2 (0.8)
Tilting of the head	14 (5.7)	130 (53.1)	94 (38.4)	5 (2.0)	2 (0.8)
Joints that are warm, swollen and sensitive, especially if child refuses weightbearing	**176 (71.8)**	2 (0.8)	63 (25.7)	2 (0.8)	2 (0.8)
Incapable of waking up a baby	**233 (95.1)**	0 (0.0)	9 (3.7)	1 (0.4)	2 (0.8)
Delay in motor development	43 (17.6)	8 (3.3)	**190 (77.6)**	190 (77.6)	1 (0.4)
Loss of sense of smell	**143 (58.4)**	1 (0.4)	82 (33.5)	16 (6.5)	3 (1.2)
Muscle weakness	56 (22.9)	15 (6.1)	**167 (68.2)**	4 (1.6)	3 (1.2)
Nystagmus	101 (41.2)	8 (3.3)	**125 (51.0)**	7 (2.9)	4 (1.6)
Pallor	97 (39.6)	8 (3.3)	**119 (48.6)**	18 (7.3)	3 (1.2)
Parental suspicion of substance abuse	**184 (75.1)**	0 (0.0)	50 (20.4)	9 (3.7)	2 (0.8)
Legg-Calve-Perthes	115 (46.9)	5 (2.0)	**117 (47.8)**	6 (2.4)	2 (0.8)
Persistent diarrhea	**157 (64.1)**	3 (1.2)	78 (31.8)	5 (2.0)	2 (0.8)
Persistent crying or faint crying with somnolence in a baby or child	**183 (74.7)**	6 (2.4)	45 (18.4)	9 (3.7)	2 (0.8)
Persistent vomiting	**208 (84.9)**	2 (0.8)	32 (13.1)	1 (0.4)	2 (0.8)
Personality change	**132 (53.9)**	3 (1.2)	99 (40.4)	9 (3.7)	2 (0.8)
Febrile petechiae or purpuric rash	**199 (81.2)**	1 (0.4)	30 (12.2)	13 (5.3)	2 (0.8)
Positive Babinski	96 (39.2)	21 (8.6)	**104 (42.4)**	21 (8.6)	3 (1,2)
Recurrent fevers	77 (31.4)	4 (1.6)	**157 (64.1)**	4 (1.6)	3 (1.2)
Redness in the nasal region	26 (10.6)	40 (16.3)	**152 (62.0)**	24 (9.8)	3 (1.2)
Nasal discharge	10 (4.1)	86 (35.1)	**137 (55.9)**	9 (3.7)	3 (1.2)
Scoliosis > 20°	36 (14.7)	6 (2.4)	**199 (81.2)**	2 (0.8)	2 (0.8)
Slipped capital femoral epiphysis	**173 (70.6)**	1 (0.4)	66 (26.9)	3 (1.2)	2 (0.8)
Signs of dehydration signs and/or a decrease of 50% of fluid intake over a 24-h period in a baby or child	**227 (92.7)**	1 (0.4)	10 (4.1)	5 (2.0)	2 (0.8)
Pressure in the sinus area	26 (10.6)	59 (24.1)	**153 (62.4)**	5 (2.0)	2 (0.8)
Speech disorders	93 (38.0)	2 (0.8)	**136 (55.5)**	12 (4.9)	2 (0.8)
Strabismus—new finding	**147 (60.0)**	3 (1.2)	82 (33.5)	11 (4.5)	2 (0.8)
Persistent abdominal pain	**189 (77.1)**	1 (0.4)	47 (19.2)	5 (2.0)	3 (1.2)
Perspiration	63 (25.7)	28 (11.4)	**117 (47.8)**	35 (14.3)	2 (0.8)
Swollen lymph nodes	79 (32.2)	25 (10.2)	**134 (54.7)**	5 (2.0)	2 (0.8)
Suicidal ideas	**197 (80.4)**	2 (0.8)	41 (16.7)	3 (1.2)	2 (0.8)
Sore throat	25 (10.2)	58 (23.7)	**149 (60.8)**	11 (4.5)	2 (0.8)
Exhaustion	20 (8.2)	45 (18.4)	**168 (68.6)**	9 (3.7)	3 (1.2)
Bruising without cause (no trauma, no abuse)	**132 (53.9)**	4 (1.6)	99 (40.4)	8 (3.3)	2 (0.8)
Unexplained weight loss	**148 (60.4)**	1 (0.4)	90 (36.7)	3 (1.2)	3 (1.2)
Watering	37 (15.1)	35 (14.3)	**149 (60.8)**	22 (9.0)	2 (0.8)
Allergies	32 (13.1)	29 (11.8)	**167 (68.2)**	15 (6.1)	2 (0.8)
Acute headache	48 (19.6)	63 (25.7)	**127 (51.8)**	4 (1.6)	3 (1.2)
Chronic headache	5 (2.0)	80 (32.7)	**155 (63.3)**	2 (0.8)	3 (1.2)
Neoplasm—circumscribed lesion	**147 (60.0)**	2 (0.8)	81 (33.1)	13 (5.3)	2 (0.8)
Neoplasm—noncircumscribed lesion	**212 (86.5)**	0 (0.0)	22 (9.0)	8 (3.3)	3 (1.2)

Bold = mode

Exclusive chiropractic care was rarely reported as the preferred management for pediatric red flags. Exclusive chiropractic care was only reported by the majority of the respondent for the management of “tilting the head”. Chiropractors commonly reported a comanagement approach to the following clinical presentations: scoliosis > 20°, allergies, delay in motor development, muscle weakness, and chest discomfort of low to medium density.

## Discussion

This research represents the first comprehensive study of current pediatric practices and trends undertaken in Quebec to collect the demographic characteristics of chiropractors who treat children. This information includes patients’ characteristics by group age as well as chiropractors’ knowledge of red flags, referral patterns, levels of educational training with respect to pediatric patients, certainty in their clinical impressions, commonly seen pediatric conditions and finally the nature of interdisciplinary collaboration. Overall, the demographics and practice characteristics of the chiropractors working Quebec included in our sample resemble those of chiropractors worldwide [4–12, 17]. Similarly to other studies, our findings suggest that Quebec chiropractors treat preadolescent and adolescent groups more frequently, followed by newborns and infants [3–12, 17]. Our participants treat a variety of conditions; similar findings have been previously reported in European, Scandinavian, Australian, American and British studies, according to which the vast majority of chiropractors focus predominantly on musculoskeletal disorders and demonstrate their confidence in their skills across all age groups [7–11, 17]. These results align with the essential core competencies established by and clinical recommendations of an expert panel focused on chiropractic pediatric patients with the expectation that chiropractors should be proficient in treating this population [21].

Furthermore, the 6–12-year-old group (57.1%) was the group most frequently treated by chiropractors in Quebec, followed by the 13–17-year-old group (54.3%). This finding may be partially explained by the fact those two groups are the groups most frequently referred by medical doctors. The scientific literature also suggests that the prevalence of MSK conditions such as back pain, headaches, sports injuries and scoliosis might be growing with age [4–12, 17, 26]. The third most frequently treated group was the 0–6-month-old group (34.7%). Our current findings differ from a Scandinavian study [8] who reported that babies aged between 0 and 3 months were the most frequent age group (58%) among pediatric patients seeking chiropractic care. However, the complaints reported in our study in this age group (torticollis, colic, excessive crying and irritability and breast-feeding problems) were similar to previous studies. This might be related to the emerging body of evidence regarding efficacy and safety of chiropractic care for pediatric MSK conditions [27–29].

The majority of respondents noted they would not hesitate to seek a medical opinion when presented with a patient who exhibited challenging signs and symptoms. Their level of confidence regarding their evaluations was legitimately lower for non-MSK conditions since these conditions had not been the focus of their training and practice. However, some chiropractors treat non-MSK conditions which are not supported by evidence. Based on a limited number of high-quality studies, a recent systematic review questioned the validity of the theory that treating spinal dysfunction with manual spinal therapy has a physiological effect on organs and their functions [30].

The results of our study indicated that the largest proportion of pediatric referrals to chiropractic services for expertise and care were from parents, siblings and other family members, followed by family physicians and nurses, with allied professionals representing the third most common source. In addition, the children most frequently referred by health professionals are those between 0–6 months and 13–18 years old. These results suggest openness to collaborative care, which might represent an opportunity to create more defined care pathways between chiropractic and medical care for pediatric patients.

Even though there is a lack of evidence regarding the effectiveness of chiropractic care for pediatric patients [30–32], some studies have suggested that the emergence of adverse effects following manual therapy is rare in this population [27–29]. It has been well documented that parents consult chiropractors regarding their children, and it is the responsibility of members of the chiropractic profession to produce methodological and high-quality studies to investigate the effectiveness of care for the benefit of pediatric patients.

### Limitations

Given our response rate, selection criteria and the higher proportion of women in our sample than among members of the OCQ in general, it is likely that our respondents are chiropractors with a particular interest in pediatric care. Therefore, our findings might not be generalizable to all chiropractors. However, we believe that this study provides particularly relevant information regarding this subgroup of chiropractors. The validity of our respondents’ answers was not assessed. Therefore, it is possible that the information reported in this study might not perfectly represent the actual clinical practices of our respondents. Although clarity issues were not highlighted during the pilot testing of our questionnaire, the formulation of the question on the confidence in diagnostic capacity might be improved by changing the answer categories to 'I am always certain', 'I am certain most of the time', 'I am certain sometimes', 'I am rarely certain', 'I am never certain'. Data was collected over a long period of time, which included the onset of the COVID-19 pandemic, and we cannot completely rule out the possibility that the profile of practice our respondent might have changed during that time.

## Conclusion

Chiropractors in Quebec are confident in their diagnoses of pediatric MSK conditions and refer patients to physicians in the rare event of a worrisome presentation. However, some chiropractors may have expectations that are unsupported by scientific evidence regarding the diagnosis and management of non-MSK complaints.

## Supplementary Information

**Additional file 1.** Survey questionnaire.

## Data Availability

The datasets analysed during the current study are available from the corresponding author on reasonable request.

## Abbreviations

AQCPP: Association québécoise de chiropratique pédiatrique et périnatal
DICCP: Diplomate in clinical chiropractic pediatric
IQR: Interquartile range
MSK: Musculoskeletal
OCQ: Ordre des chiropraticiens du Québec
UQTR: Université du Québec à Trois-Rivières
