# Supplementary material for: Chiropractic pediatric patient management and interdisciplinary collaboration: a descriptive cross-sectional study of chiropractors in Quebec
Source: Chiropr Man Therap. 2022 Dec 13;30:54. doi: 10.1186/s12998-022-00464-y (PMC9746577; doi:10.1186/s12998-022-00464-y)
Supplement: Supplementary file 1 — Additional file 1. Survey questionnaire. [file 12998_2022_464_MOESM1_ESM.pdf]

1. Consentement au sondage

Université du Québec à Trois-Rivières

Titre de l'étude :
Étude descriptive transversale : La pratique courante des chiropraticiens québécois en regard de la pédiatrie – PCDCQ – Pédiatrie

Équipe de recherche :
Chantal Doucet, professeure-clinicienne superviseure
Camille Imbeau, interne senior au programme de doctorat de premier cycle

Introduction et Objectif :
L'objectif principal de ce projet de recherche vise à documenter la pratique courante des chiropraticiens québécois auprès de la population pédiatrique. Pour ce faire, l'équipe de recherche a adapté un questionnaire préexistant afin de mieux refléter la réalité de la pratique chiropratique québécoise.

Ce que vous devez faire :
Vous êtes sollicités pour répondre à ce questionnaire en ligne, et ce sur une base volontaire. Cela implique que vous aurez à répondre à certaines dimensions de votre pratique courante concernant votre clientèle pédiatrique actuelle. Le déroulement du questionnaire devrait prendre tout au plus 30 minutes à compléter. Pour que toutes vos réponses soient bien enregistrées, vous devez vous rendre à la fin du questionnaire et cliquer sur ''**Terminé**'' au bas de la page. Cette disposition démontrera votre plein consentement à la participation de ce questionnaire en ligne.

Risques/Bénéfices:
Les risques du répondant à participer à ce projet sont minimes. La collecte de données et la réponse au questionnaire comportent les mêmes risques que l'utilisation quotidienne de l'Internet, tel qu'une violation de la confidentialité. Bien que les chercheurs aient pris toutes les mesures raisonnables pour protéger votre confidentialité, il existe toujours un risque d'interception du piratage des données par des tiers qui ne soit pas sous le contrôle de l'équipe de recherche. Il n'y a aucun avantage pour vous directement, excepté de potentiellement contribuer aux connaissances du profil de pratique et de la gestion clinique de la population pédiatrique en chiropratique au Québec.

Confidentialité:
Ce sondage est anonyme et nous ne collecterons pas d'informations qui permettraient de vous identifier facilement, telles que votre nom ou d'autres identifiants uniques. Bien que votre adresse IP puisse être suivie via la plateforme d'enquête, les chercheurs ne collecteront pas ces informations. Votre adresse IP peut être observée uniquement pour nous assurer qu'une personne ne remplit pas le sondage plusieurs fois.
Les données seront conservées sur le site web de SurveyMonkey pendant douze mois et seront ensuite supprimées par le personnel de recherche. Cependant, des données peuvent exister sur les sauvegardes au-delà de la période visée par ce projet de recherche. Les données transférées à partir du site de sondage seront sauvegardées par le PI sur un ordinateur protégé par mot de passe pendant une période maximale de 7 ans. Seul le personnel de recherche aura accès aux données recueillies par cette étude. Ce projet de recherche a fait l'objet d'une analyse et conséquemment d'une acceptation par le Comité de programme du département de chiropratique de l'université de Québec à Trois-Rivières.

Incitation à la participation:
Vous ne serez pas rémunéré pour votre participation à cette étude.

Vos droits en tant que participants à la recherche:

La participation à cette recherche est entièrement volontaire et vous pouvez retirer votre consentement à tout moment en cliquant sur le bouton ‘’Terminé’’ situé à la fin du sondage. Toutefois, comme le sondage est anonyme, une fois que vous avez cliqué sur le bouton ‘’Terminé’’ à la fin du sondage, les chercheurs ne pourront pas déterminer quel est le sondage qui vous appartient. Ainsi, vos informations ne peuvent plus être retirées après ce moment.

Qui dois-je contacter pour des questions sur l'étude:

Pour plus d'informations sur les procédures de l'étude, contactez le superviseur Chantal Doucet au 819-376-5011 poste 4471.

Qui dois-je contacter pour des questions sur mes droits ou des plaintes concernant mon traitement en tant que sujet de recherche?

Contactez le superviseur Chantal Doucet au 819-376-5011 poste 4471.

Lien vers la Politique de confidentialité de SurveyMonkey: https://fr.surveymonkey.com/mp/legal/privacy-policy/

* 1. En participant à cette enquête, vous indiquez que vous avez lu le formulaire de consentement et que vous avez 18 ans ou plus.

- ○ Continuer
- ○ Terminer

2. Données démographiques

* 2. Êtes-vous :

- [ ] Une femme
- [ ] Un homme
- [ ] Je préfère ne pas répondre
- [ ] Autre (veuillez préciser)

* 3. Pratiquez-vous présentement au Québec?

- ○ Oui
- ○ Non

* 4. Depuis combien de temps pratiquez-vous (en année)? Veuillez indiquer le nombre dans la case suivante.

* 5. Traitez-vous des enfants âgés de moins de 18 ans (0-17 ans)?

- ○ Oui
- ○ Non

3. Profil de la pratique courante en pédiatrie

En rétrospective du DERNIER mois de votre pratique:

* 6. En moyenne, combien de **patients pédiatriques** (0-17 ans) traitez-vous ou voyez-vous en **une semaine** (nombre de patients)? (Toutes les visites, incluant les nouveaux patients)

- ◯ 0-5 patients
- ◯ 6-10 patients
- ◯ 11-15 patients
- ◯ 16-20 patients
- ◯ 21 patients et plus

* 7. En moyenne, combien de **nouveaux patients pédiatriques** traitez-vous ou voyez-vous en **une semaine**?
Un nouveau patient pédiatrique (0-17 ans) est défini comme un patient arrivant à votre clinique pour la première fois, ou un patient actuel arrivant avec une nouvelle problématique.

- ◯ 0-5 patients
- ◯ 6-10 patients
- ◯ 11-15 patients
- ◯ 16-20 patients
- ◯ 21 patients et plus

* 8. Veuillez sélectionner **les DEUX groupes d'âge** qui vous consultent le plus fréquemment (nombre de patient d'un groupe d'âge **par semaine**.)

- ☐ 0-6 mois
- ☐ 7-23 mois
- ☐ 2-5 ans
- ☐ 6-12 ans
- ☐ 13-17 ans

* 9. Quel est le type de votre pratique?

- ◯ Pratique solo (vous êtes le/la seul(e) chiropraticien(ne) dans votre clinique)
- ◯ Pratique en groupe de chiropraticiens (vous êtes deux chiropraticiens ou plus dans la même clinique)
- ◯ Pratique multidisciplinaire (DC/MD)
- ◯ Pratique multidisciplinaire (DC/ médecine alternative et complémentaire (MAC))

* 10. Quel est l'objectif de votre traitement chiropratique? Veuillez sélectionner le choix qui répond le mieux à votre situation.

- ◯ Améliorer la fonction
- ◯ Diminuer la douleur
- ◯ Prévention
- ◯ Autre (veuillez préciser)
- ◯ Éliminer les subluxations
- ◯ Améliorer les habitudes de vies
- ◯ Améliorer la qualité de vie

4. Provenance de votre référencement

* 11. Les patients pédiatriques sont référés à votre clinique de différentes façons.
Veuillez identifier **tous** les moyens de référence des patients pédiatriques à votre clinique ci-dessous:

- [ ] Référence d'un pédiatre
- [ ] Référence d'un hôpital pédiatrique
- [ ] Référence d'un physiothérapeute (pédiatrique)
- [ ] Référence d'orthophoniste
- [ ] Référence d'ergothérapeute
- [ ] Référence d'un psychologue
- [ ] Référence d'un médecin de famille
- [ ] Référence d'un médecin spécialiste ne travaillant pas à l'hôpital
- [ ] Référence d'autres chiropraticiens
- [ ] Référence d'une infirmière
- [ ] Référence d'une sage-femme
- [ ] Référence d'un nutritionniste
- [ ] Référence d'un naturopathe
- [ ] Référence de parents / fratrie ou autres membres de la famille du patient
- [ ] Référence par bouche à oreille / Référence hors-famille du patient (collègue, voisin, etc)
- [ ] Référence de l'Association québécoise de chiropratique pédiatrique et en périnatalité (AQCPP)
- [ ] Référence de l'Association des chiropraticiens du Québec (ACQ)
- [ ] Référence de l'Ordre des chiropraticiens du Québec (OCQ)
- [ ] Référence par publicité (Site web, article de journal, Journée de dépistage, Conférences et salons, etc.)
- [ ] Autre (veuillez préciser)

* 12. Veuillez sélectionner les **<u>deux (2)</u>** modes de références les plus fréquents dans votre clinique concernant vos patients pédiatriques:

- ☐ Référence d'un pédiatre
- ☐ Référence d'un hôpital pédiatrique
- ☐ Référence d'un physiothérapeute (pédiatrique)
- ☐ Référence d'orthophoniste
- ☐ Référence d'ergothérapeute
- ☐ Référence d'un psychologue
- ☐ Référence d'un médecin de famille
- ☐ Référence d'un médecin spécialiste ne travaillant pas à l'hôpital
- ☐ Référence d'autres chiropraticiens
- ☐ Référence d'une infirmière (CLSC)
- ☐ Référence d'une sage-femme
- ☐ Référence d'un nutritionniste
- ☐ Référence d'un naturopathe
- ☐ Référence de parents / fratrie ou autres membres de la famille du patient
- ☐ Référence par bouche à oreille / Référence hors-famille du patient (collègue, voisin, etc)
- ☐ Référence de l'Association québécoise de chiropratique pédiatrique et en périnatalité (AQCPP)
- ☐ Référence de l'Association des chiropraticiens du Québec (ACQ)
- ☐ Référence de l'Ordre des chiropraticiens du Québec (OCQ)
- ☐ Référence par publicité (Site web, article de journal, Journée de dépistage, Conférences et salons, etc.)
- ☐ Autre (veuillez préciser)

* 13. De tous les patients pédiatriques référés par des pédiatres ou médecins de famille, quels sont les **<u>deux (2)</u>** groupes d'âge les plus fréquents que vous voyez?

- ☐ 0-6 mois
- ☐ 7-23 mois
- ☐ 2-5 ans
- ☐ 6-12 ans
- ☐ 13-17 ans

* 14. À quelle fréquence, en moyenne, référez-vous vos patients pédiatriques à une infirmière clinicienne ou un médecin de famille (GMF, clinique de périnatalité)?

- ◯ Jamais
- ◯ Rarement (<1 fois/mois)
- ◯ Quelques fois (1-3 fois/mois)
- ◯ Souvent (1-2 fois/semaine)
- ◯ Fréquemment (>2 fois/semaine)

* 15. À quelle fréquence, en moyenne, référez-vous vos patients pédiatriques à un pédiatre ou un autre spécialiste pédiatrique?

- ◯ Jamais
- ◯ Rarement (<1 fois/mois)
- ◯ Quelques fois (1-3 fois/mois)
- ◯ Souvent (1-2 fois/semaine)
- ◯ Fréquemment (>2 fois/semaine)

5. Pratique interdisciplinaire avec d'autres professionnels de la santé pédiatrique

* 16. À quelle fréquence communiquez-vous par écrit (lettre de cogestion, référence pour un deuxième avis, demande d'investigation, etc) au sujet de vos patients pédiatriques à leur pédiatre?

- ◯ Jamais
- ◯ Rarement (<1 /mois)
- ◯ Quelques fois (1-3/mois)
- ◯ Souvent (1-2/semaine)
- ◯ Fréquemment (>2/semaine)

* 17. Les professionnels en périnatalité et en santé pédiatrique faisant partie du **réseau de la santé** comprennent les pédiatres, les médecins de familles, les infirmières, les ergothérapeutes, les physiothérapeutes, les pharmaciens, les dentistes, les psychologues et les nutritionnistes.

En moyenne, enseignez/informez-vous, ou avez-vous déjà enseigné/informé sur les soins chiropratiques concernant la clientèle pédiatrique à un ou plusieurs de ces professionnels?

- ◯ Jamais
- ◯ Rarement (1-2 fois en tout)
- ◯ Occasionnellement (1 fois/2 à 3 ans)
- ◯ Souvent (1 fois/an)
- ◯ Fréquemment (Plus d'une fois/an)

* 18. Les professionnels en périnatalité et en santé pédiatrique faisant partie du **consortium des médecines alternatives complémentaires (MAC)** comprennent, entre autres, les massothérapeuthes, les naturopathes, les acupuncteurs et les ostéopathes, et ceux de la profession paramédicale gravitant autour de la dyade mère-enfant incluent les sages-femmes, les IBCLC (consultantes en lactation), les accompagnantes à l'accouchement et les marraines d'allaitement.

En moyenne, enseignez/informez-vous, ou avez-vous déjà enseigné/informé sur les soins chiropratiques concernant la clientèle pédiatrique à un ou plusieurs de ces professionnels?

- ◯ Jamais
- ◯ Rarement (1-2 fois en tout)
- ◯ Occasionnellement (1 fois / 2 à 3 ans)
- ◯ Souvent (1 fois/an)
- ◯ Fréquemment (Plus d'une fois /an)

* 19. Êtes-vous invité, ou avez-vous été invité, à participer à un congrès médical, à un séminaire, une présentation à un GMF (Groupe de médecine familiale), à un groupe de médecin, une réunion départementale médicale ou des ateliers thématiques dans des CLSC pour représenter la chiropratique et ses opportunités auprès de la population pédiatrique?

- ◯ Jamais
- ◯ Rarement (1-2 fois en tout)
- ◯ Occasionnellement (1 fois / 2 à 3 ans)
- ◯ Souvent (1 fois /an)
- ◯ Fréquemment (Plus d'une fois/an)

6. Formation continue en pédiatrie

* 20. Auxquelles de ces formations continues en pédiatrie participez-vous? Veuillez identifier toutes celles qui s’appliquent :

- [ ] Lecture de publications scientifiques
- [ ] Participer à des séminaires ou conférences de chiropratique/médecine/nutritionniste/diététiste/sage-femme sur des thèmes tels que l'allaitement, le traitement de la plagiocéphalie ou du torticolis congénital
- [ ] Participer à de la formation continue dans un hôpital pédiatrique
- [ ] Participer à des cours de formation post-gradué en pédiatrie
- [ ] Participer à des formations de pédiatrie données par l'Association Québécoise de Chiropratique en Périnatalité et Pédiatrie (AQCPP)
- [ ] Participer à des formations de pédiatrie données par l'Ordre des Chiropraticiens du Québec (OCQ)
- [ ] Autre (veuillez préciser)

* 21. En moyenne, combien d’heures de formation continue faites-vous, par année, en pédiatrie?
Veuillez indiquer le nombre d'heures dans la case ci-dessous.

* 22.
Diagnostic clinique

Nous cherchons à connaitre votre niveau de certitude en regard du diagnostic des catégories suivantes.

Pour chaque condition suivante, veuillez indiquer si vous n’êtes pas du tout d’accord, pas d’accord, ni en désaccord ni en accord, d’accord ou tout à fait d’accord.

	Pas du tout d’accord	Pas d’accord	Ni en désaccord ni en accord	D’accord	Tout à fait d’accord
Troubles musculosquelettiques chez les nouveau-nés	○	○	○	○	○
Troubles musculosquelettiques chez les enfants d’âge pré-scolaire	○	○	○	○	○
Troubles musculosquelettiques chez les enfants d’âge scolaire (école primaire)	○	○	○	○	○
Troubles musculosquelettiques chez les enfants d’âge scolaire (école secondaire)	○	○	○	○	○
Troubles non musculosquelettiques chez les nouveau-nés	○	○	○	○	○
Troubles non musculosquelettiques chez les enfants d’âge pré-scolaire	○	○	○	○	○
Troubles non musculosquelettiques chez les enfants d’âge scolaire (école primaire)	○	○	○	○	○
Troubles non musculosquelettiques chez les enfants d’âge scolaire (école secondaire)	○	○	○	○	○

7. Conditions cliniques en pédiatrie

* 23. Dans la dernière année, **combien de fois** par semaine, en moyenne, avez-vous donné les conseils suivants à vos patients pédiatriques? (Vous avez pu donner plus d'un conseil au même patient.)

	Jamais	Rarement (1 x/4 semaines)	Quelques fois (1 x/2 semaines)	Souvent (2 x/semaine)	Fréquemment (5 x/semaine)
Recommandations d'allaitement	○	○	○	○	○
Changements d'habitudes à risque ou malsaines	○	○	○	○	○
Prévention de maladie ou conseils de dépistage (ex: scoliose)	○	○	○	○	○
Recommandations nutritionnelles	○	○	○	○	○
Promouvoir l'activité physique	○	○	○	○	○
Recommandations de relaxation ou de diminution du stress	○	○	○	○	○
Stratégies de soins à la maison	○	○	○	○	○

Autre (veuillez préciser)

* 24. Dans le groupe d’âge 0-6 mois, quelles sont les conditions que vous traitez? Veuillez **cocher <u>toutes les boites</u>** qui s’appliquent (incluant, de façon générale, tous les motifs de consultation).

- [ ] Torticolis infantile
- [ ] Utilisation d'instruments à l'accouchement
- [ ] Asymétrie de la tête/Plagiocéphalie/Brachycéphalie
- [ ] Mouvements anormaux
- [ ] Coliques infantiles/Pleurs excessifs/Irritabilité
- [ ] Problèmes digestifs/Estomac/ Reflux gastroœsophagien
- [ ] Problèmes d’allaitement/ (Dysfonction de la succion, prise su sein, position allaitement)
- [ ] Problème de sommeil
- [ ] Douleur aux oreilles/ Otite moyenne
- [ ] Dysplasie de la hanche
- [ ] Examen prophylactique
- [ ] Allergies aux protéines bovines
- [ ] Bronchiolite
- [ ] Bébé hypertonique
- [ ] Paralysie obstétricale du plexus brachial
- [ ] Problème de mâchoire
- [ ] Jaunisse
- [ ] Fièvre
- [ ] Retard du développement moteur
- [ ] Autre (veuillez préciser)

* 25. Dans le groupe d’âge 0-6 mois, quelles sont les **DEUX (2)** conditions de motif de consultation les plus fréquentes à votre clinique? Veuillez **cocher les deux boites** qui s’appliquent.

- [ ] Torticolis infantile
- [ ] Utilisation d'instruments à l'accouchement
- [ ] Asymétrie de la tête/Plagiocéphalie/Brachycéphalie
- [ ] Mouvements anormaux
- [ ] Coliques infantiles/Pleurs excessifs/Irritabilité
- [ ] Problèmes digestifs/Estomac/ Reflux gastroœsophagien
- [ ] Problèmes d’allaitement/ (Dysfonction de la succion, prise su sein, position allaitement)
- [ ] Problème de sommeil
- [ ] Douleur aux oreilles/ Otite moyenne
- [ ] Dysplasie de la hanche
- [ ] Examen prophylactique
- [ ] Allergies aux protéines bovines
- [ ] Bronchiolite
- [ ] Bébé hypertonique
- [ ] Paralysie obstétricale du plexus brachial
- [ ] Problème de mâchoire
- [ ] Jaunisse
- [ ] Fièvre
- [ ] Retard du développement moteur
- [ ] Autre (veuillez préciser)

* 26. Dans le groupe d’âge 0-6 mois, quelles sont les conditions que vous traitez **le plus souvent**, en terme de fréquence (ne sélectionner que **2 cases**)?

- ☐ Asymétrie de la tête/Plagiocéphalie/Brachycéphalie
- ☐ Mouvements anormaux
- ☐ Utilisation d'instruments à l'accouchement
- ☐ Coliques infantiles/Pleurs excessifs/Irritabilité
- ☐ Problèmes digestifs/Estomac/ Reflux gastroœsophagien
- ☐ Problèmes d’allaitement/ (Dysfonction de la succion, prise du sein, position d'allaitement)
- ☐ Problème de sommeil
- ☐ Douleur aux oreilles/ Otite moyenne
- ☐ Autre (veuillez préciser)
- ☐ Examen prophylactique
- ☐ Bronchiolite
- ☐ Bébé hypertonique
- ☐ Paralysie obstétricale du plexus brachial
- ☐ Jaunisse
- ☐ Fièvre
- ☐ Retard du développement moteur

* 27. Dans le groupe d’âge 7-23 mois, quelles sont les conditions que vous traitez? Veuillez cocher **toutes les boites** qui s’appliquent (incluant les motifs primaires et secondaires).

- ☐ Asymétrie/Torticolis infantile
- ☐ Asymétrie de la tête/Plagiocéphalie/Brachycéphalie
- ☐ Mouvements anormaux
- ☐ Coliques infantiles/Pleurs excessifs/Irritabilité
- ☐ Problèmes digestifs/Estomac/ Reflux gastroœsophagien
- ☐ Problème de sommeil
- ☐ Douleur aux oreilles/ Otite moyenne
- ☐ Allergie
- ☐ Autre (veuillez préciser)
- ☐ Troubles de la marche
- ☐ Examen prophylactique
- ☐ Asthme
- ☐ Bronchite
- ☐ Otite séreuse
- ☐ Autisme
- ☐ Trouble envahissant du comportement

* 28. Dans le groupe d’âge 7-23 mois, quelles sont les **DEUX (2)** conditions de motif de consultation les plus fréquentes à votre clinique? Veuillez **cocher les deux boites** qui s’appliquent.

- [ ] Asymétrie/Torticolis infantile
- [ ] Asymétrie de la tête/Plagiocéphalie/Brachycéphalie
- [ ] Mouvements anormaux
- [ ] Coliques infantiles/Pleurs excessifs/Irritabilité
- [ ] Problèmes digestifs/Estomac/ Reflux gastroœsophagien
- [ ] Problème de sommeil
- [ ] Douleur aux oreilles/ Otite moyenne
- [ ] Allergie
- [ ] Autre (veuillez préciser)
- [ ] Troubles de la marche
- [ ] Examen prophylactique
- [ ] Asthme
- [ ] Bronchite
- [ ] Otite séreuse
- [ ] Autisme
- [ ] Trouble envahissant du comportement

* 29. Dans le groupe d’âge 7-23 mois, quelles sont les **DEUX (2)** conditions que vous traitez ou voyez **le plus souvent**, en terme de fréquence? Veuillez cocher **les deux boites** qui s’appliquent.

- [ ] Asymétrie/Torticolis infantile
- [ ] Asymétrie de la tête/Plagiocéphalie/Brachycéphalie
- [ ] Mouvements anormaux
- [ ] Coliques infantiles/Pleurs excessifs/Irritabilité
- [ ] Problèmes digestifs/Estomac/ Reflux gastroœsophagien
- [ ] Problème de sommeil
- [ ] Douleur aux oreilles/ Otite moyenne
- [ ] Allergie
- [ ] Autre (veuillez préciser)
- [ ] Troubles de la marche
- [ ] Examen prophylactique
- [ ] Asthme
- [ ] Bronchite
- [ ] Otite séreuse
- [ ] Autisme
- [ ] Trouble envahissant du comportement

* 30. Dans le groupe d’âge 2-5 ans, quelles sont les conditions que vous traitez ou voyez? Veuillez **cocher toutes** les boites qui s’appliquent (incluant les motifs primaires et secondaires).

- [ ] Torticolis infantile (non résolu) / Asymétrie
- [ ] Asymétrie de la tête /plagiocéphalie/Brachycéphalie
- [ ] Développement moteur
- [ ] Plainte musculosquelettique / douleurs à la colonne cervicale
- [ ] Plainte musculosquelettique / douleurs à la colonne thoracique
- [ ] Plainte musculosquelettique / douleurs à la colonne lombaire, bassin
- [ ] Plainte musculosquelettique / douleurs aux extrémités supérieures
- [ ] Plainte musculosquelettique / douleurs aux extrémités inférieures
- [ ] Posture
- [ ] Maux de tête/Syndrome pré-migraineux
- [ ] Chutes
- [ ] Autre (veuillez préciser)
- [ ] Douleur de croissance
- [ ] Douleur à l’oreille/otite moyenne
- [ ] Asthme/allergie
- [ ] Examen prophylactique (prévention)
- [ ] Fièvre
- [ ] Otite séreuse
- [ ] Bronchite
- [ ] Pneumonie
- [ ] Scoliose
- [ ] Énurésie nocturne

* 31. Dans le groupe d’âge 2-5 ans, quelles sont les **deux (2) conditions** de motif de consultation les plus fréquentes à votre clinique? Veuillez **cocher les deux boites** qui s’appliquent.

- [ ] Torticolis infantile (non résolu) / Asymétrie
- [ ] Asymétrie de la tête/Plagiocéphalie/Brachycéphalie
- [ ] Développement moteur
- [ ] Plainte musculosquelettique / douleur à la colonne cervicale
- [ ] Plainte musculosquelettique / douleur à la colonne thoracique
- [ ] Plainte musculosquelettique / douleur à la colonne lombaire, bassin
- [ ] Plainte musculosquelettique / douleurs aux extrémités supérieures
- [ ] Plainte musculosquelettique / douleurs aux extrémités inférieures
- [ ] Posture
- [ ] Maux de tête/Syndrome pré-migraineux
- [ ] Blessure traumatique
- [ ] Autre (veuillez préciser)
- [ ] Douleur de croissance
- [ ] Douleur à l'oreille/otite moyenne
- [ ] Asthme/Allergie
- [ ] Examen prophylactique (prévention)
- [ ] Fièvre
- [ ] Otite séreuse
- [ ] Bronchite
- [ ] Pneumonie
- [ ] Scoliose
- [ ] Énurésie nocturne

* 32. Dans le groupe d’âge 2-5 ans, quelles sont les **DEUX (2)** conditions que vous traitez ou voyez **le plus souvent**, en terme de fréquence? Veuillez cocher **les deux boites** qui s’appliquent.

- ☐ Torticolis infantile (non résolu) / Asymétrie
- ☐ Asymétrie de la tête /plagiocéphalie/Brachycéphalie
- ☐ Développement moteur
- ☐ Plainte musculosquelettique / douleur à la colonne cervicale
- ☐ Plainte musculosquelettique / douleur à la colonne thoracique
- ☐ Plainte musculosquelettique / douleur à la colonne lombaire, bassin
- ☐ Plainte musculosquelettique / douleurs aux extrémités supérieures
- ☐ Plainte musculosquelettique / douleurs aux extrémités inférieures
- ☐ Posture
- ☐ Maux de tête/Syndrome pré-migraineux
- ☐ Chutes
- ☐ Autre (veuillez préciser)
- ☐ Douleur de croissance
- ☐ Douleur à l’oreille/otite moyenne
- ☐ Asthme/allergie
- ☐ Examen prophylactique (prévention)
- ☐ Fièvre
- ☐ Otite séreuse
- ☐ Bronchite
- ☐ Pneumonie
- ☐ Scoliose
- ☐ Énurésie nocturne

* 33. Dans le groupe d’âge 6-12 ans, quelles sont les conditions que vous traitez ou voyez? Veuillez **cocher toutes les boites** qui s’appliquent (incluant les motifs primaires et secondaires).

- ☐ Développement moteur
- ☐ Plainte musculosquelettique / douleurs à la colonne cervicale
- ☐ Plainte musculosquelettique / douleur à la colonne thoracique
- ☐ Plainte musculosquelettique / douleur à la colonne lombaire, bassin
- ☐ Plainte musculosquelettique / douleurs aux extrémités supérieures
- ☐ Plainte musculosquelettique / douleurs aux extrémités inférieures
- ☐ Posture
- ☐ Scoliose
- ☐ Migraines
- ☐ Autre (veuillez préciser)
- ☐ Blessure traumatique
- ☐ Blessures sportives
- ☐ Douleur de croissance
- ☐ Douleur à l’oreille/otite séreuse
- ☐ Asthme/allergie
- ☐ Concentration/hyperactivité
- ☐ Troubles du sommeil
- ☐ Examen prophylactique (prévention)
- ☐ Profil psychologique

* 34. Dans le groupe d’âge 6-12 ans, quelles sont **les DEUX (2)** conditions de motif de consultation les plus fréquentes à votre clinique? Veuillez cocher **les deux boites** qui s’appliquent.

- [ ] Développement moteur
- [ ] Plainte musculosquelettique / douleurs à la colonne cervicale
- [ ] Plainte musculosquelettique / douleur à la colonne thoracique
- [ ] Plainte musculosquelettique / douleur à la colonne lombaire, bassin
- [ ] Plainte musculosquelettique / douleurs aux extrémités supérieures
- [ ] Plainte musculosquelettique / douleurs aux extrémités inférieures
- [ ] Posture
- [ ] Scoliose
- [ ] Migraines
- [ ] Autre (veuillez préciser)
- [ ] Blessure traumatique
- [ ] Blessures sportives
- [ ] Douleur de croissance
- [ ] Douleur à l’oreille/otite séreuse
- [ ] Asthme/allergie
- [ ] Concentration/hyperactivité
- [ ] Troubles du sommeil
- [ ] Examen prophylactique (prévention)
- [ ] Profil psychologique

* 35. Dans le groupe d’âge 6-12 ans, quelles sont **les DEUX (2)** conditions que vous traitez ou voyez le plus souvent, en terme de fréquence? Veuillez cocher **les deux boites** qui s’appliquent.

- [ ] Développement moteur
- [ ] Plainte musculosquelettique / douleurs à la colonne cervicale
- [ ] Plainte musculosquelettique / douleur à la colonne thoracique
- [ ] Plainte musculosquelettique / douleur à la colonne lombaire, bassin
- [ ] Plainte musculosquelettique / douleurs aux extrémités supérieures
- [ ] Plainte musculosquelettique / douleurs aux extrémités inférieures
- [ ] Posture
- [ ] Scoliose
- [ ] Migraines
- [ ] Autre (veuillez préciser)
- [ ] Blessure traumatique
- [ ] Blessures sportives
- [ ] Douleur de croissance
- [ ] Douleur à l’oreille/otite séreuse
- [ ] Asthme/allergie
- [ ] Concentration/hyperactivité
- [ ] Troubles du sommeil
- [ ] Examen prophylactique (prévention)
- [ ] Profil psychologique

* 36. Dans le groupe d'âge 13-17 ans, quelles sont les conditions que vous traitez ou voyez? Veuillez **cocher toutes les boites** qui s'appliquent (incluant les motifs primaires et secondaires).

- ☐ Plainte musculosquelettique / colonne cervicale
- ☐ Plainte musculosquelettique / colonne thoracique
- ☐ Plainte musculosquelettique /colonne lombaire, bassin
- ☐ Plainte musculosquelettique / extrémités
- ☐ Posture
- ☐ Maladie de Scheuermann's
- ☐ Scoliose
- ☐ Maux de tête
- ☐ Étourdissements
- ☐ Autre (veuillez préciser)
- ☐ Blessure due au sport
- ☐ Blessure traumatique
- ☐ Concentration/hyperactivité
- ☐ Asthme/allergie
- ☐ Menstruation
- ☐ Problème de sommeil
- ☐ Examen prophylactique (prévention)
- ☐ Conditions psychologiques
- ☐ Troubles du comportement

* 37. Dans le groupe d'âge 13-17 ans, quelles sont les **DEUX (2)** conditions de motif de consultation les plus fréquentes à votre clinique? Veuillez **cocher les deux** boites qui s'appliquent.

- ☐ Plainte musculosquelettique / colonne cervicale
- ☐ Plainte musculosquelettique / colonne thoracique
- ☐ Plainte musculosquelettique /colonne lombaire, bassin
- ☐ Plainte musculosquelettique / extrémités
- ☐ Posture
- ☐ Maladie de Scheuermann's
- ☐ Scoliose
- ☐ Maux de tête
- ☐ Étourdissements
- ☐ Autre (veuillez préciser)
- ☐ Blessure due au sport
- ☐ Blessure traumatique
- ☐ Concentration/hyperactivité
- ☐ Asthme/allergie
- ☐ Menstruation
- ☐ Problème de sommeil
- ☐ Examen prophylactique (prévention)
- ☐ Conditions psychologiques
- ☐ Troubles du comportement

* 38. Dans le groupe d'âge 13-17 ans, quelles sont les **DEUX (2)** conditions que vous traitez ou voyez **le plus souvent**, en terme de fréquence? Veuillez cocher **les deux boites** qui s'appliquent.

- [ ] Plainte musculosquelettique / colonne cervicale
- [ ] Plainte musculosquelettique / colonne thoracique
- [ ] Plainte musculosquelettique /colonne lombaire, bassin
- [ ] Plainte musculosquelettique / extrémités
- [ ] Posture
- [ ] Maladie de Scheuermann's
- [ ] Scoliose
- [ ] Maux de tête
- [ ] Autre (veuillez préciser)
- [ ] Étourdissements
- [ ] Blessure due au sport
- [ ] Blessure traumatique
- [ ] Concentration/hyperactivité
- [ ] Asthme/allergie
- [ ] Menstruation
- [ ] Problème de sommeil
- [ ] Examen prophylactique (prévention)

8. Conditions cliniques particulières

* 39. Veuillez indiquer, pour chacun des signes et symptômes suivants présents chez un patient pédiatrique, la réponse qui vous semble la plus appropriée.

	Référence immédiate à l'hôpital	Soins chiropratiques seulement	Co-gestion	Je préfère ne pas répondre
Absence des réflexes primitifs	○	○	○	○
Perte de poids récente de plus de 5% du poids corporel d'un bébé ou bambin	○	○	○	○
État mental altéré, signes de déshydratation, douleur abdominale ou "haleine fruitée" d'un enfant diabétique	○	○	○	○
Ataxie	○	○	○	○
Vomissements teinté de bile	○	○	○	○
Fontanelle bombée ou creuse chez un bébé ou bambin	○	○	○	○
Fracture osseuse ou dislocation	○	○	○	○
Inconfort de la poitrine de basse à moyenne intensité	○	○	○	○
Extrémités inférieures froides ou blanches et/ou cyanose buccale	○	○	○	○
Convulsions, particulièrement si aucun historique antérieur ou associé à des traumas crâniens	○	○	○	○
Étourdissements	○	○	○	○
Dyspnée, pouvant être accompagnée de battements des ailes du nez ou d'une augmentation significative du rythme respiratoire	○	○	○	○

* 40. Veuillez indiquer, pour chacun des signes et symptômes suivants présents chez un patient pédiatrique, la réponse qui vous semble la plus appropriée.

	Référence immédiate à l'hôpital	Soins chiropratiques seulement	Co-gestion	Je préfère ne pas répondre
Sang dans les selles	◯	◯	◯	◯
Fièvre de plus de 38 degrés Celcius (prise rectale) chez un enfant âgés de plus de 90 jours	◯	◯	◯	◯
Fièvre, douleur à la poitrine, état mental altéré ou autre trouvailles neurologiques	◯	◯	◯	◯
Fièvre égale ou supérieurement à 40 degrés Celcius, particulièrement par augmentation rapide	◯	◯	◯	◯
Inclinaison de la tête	◯	◯	◯	◯
Articulations chaudes, enflées et sensibles, spécialement si l'enfant refuse de supporter son poids	◯	◯	◯	◯
Incapacité de réveiller un bébé ou bambin	◯	◯	◯	◯
Retard de développement neuromoteur	◯	◯	◯	◯
Perte de l'odorat	◯	◯	◯	◯
Faiblesse musculaire	◯	◯	◯	◯
Nystagmus	◯	◯	◯	◯
Pâleur	◯	◯	◯	◯
Suspicion des parents d'un abus de substance chimique	◯	◯	◯	◯
Maladie de Legg-Calve-Perthes	◯	◯	◯	◯
Diarrhée persistante	◯	◯	◯	◯
Pleurs persistants et haut perchés ou faibles cris avec somnolence chez un bébé ou un bambin	◯	◯	◯	◯

	Référence immédiate à l'hôpital	Soins chiropratiques seulement	Co-gestion	Je préfère ne pas répondre
Vomissements persistants	◯	◯	◯	◯
Changements dans la personnalité	◯	◯	◯	◯
Purpura fébrile avec pétéchies	◯	◯	◯	◯
Babinski positif	◯	◯	◯	◯

Autres/Commentaires (veuillez préciser)

* 41. Veuillez indiquer, pour chacun des signes et symptômes suivants présents chez un patient pédiatrique, la réponse qui vous semble la plus appropriée.

	Référence immédiate à l'hôpital	Soins chiropratiques seulement	Co-gestion	Je préfère ne pas répondre
Fièvres récurrentes	◯	◯	◯	◯
Rougeurs dans la région nasale	◯	◯	◯	◯
Écoulements nasaux	◯	◯	◯	◯
Scoliose de plus de 20 degrés	◯	◯	◯	◯
Glissement épiphysaire fémoral	◯	◯	◯	◯
Signes de déshydratation et/ou diminution de l'ingestion de fluide de 50% ou plus sur une période de 24 heures chez un bébé ou un bambin	◯	◯	◯	◯
Pression au niveau des sinus	◯	◯	◯	◯
Troubles d'élocution	◯	◯	◯	◯
Strabisme - nouvelle apparition	◯	◯	◯	◯
Soudaine apparition de symptômes de douleurs abdominales persistantes	◯	◯	◯	◯
Transpiration	◯	◯	◯	◯
Ganglions lymphatiques enflés	◯	◯	◯	◯

	Référence immédiate à l'hôpital	Soins chiropratiques seulement	Co-gestion	Je préfère ne pas répondre
Idées suicidaires	◯	◯	◯	◯
Irritation de la gorge	◯	◯	◯	◯
Fatigue	◯	◯	◯	◯
Ecchymose non-expliquée, sans trauma ni suspicion de maltraitance	◯	◯	◯	◯
Changements dans la personnalité	◯	◯	◯	◯
Perte de poids non expliquée	◯	◯	◯	◯
Larmoiement	◯	◯	◯	◯
Allergies	◯	◯	◯	◯
Céphalée aigue	◯	◯	◯	◯
Céphalée chronique	◯	◯	◯	◯
Néoplasie - lésion bien délimitée	◯	◯	◯	◯
Néoplasie - lésion mal délimitée	◯	◯	◯	◯

Autres/Commentaires (veuillez préciser)

[ ]

9. Profil professionnel

* 42. Êtes-vous membre d'une organisation ou d'un regroupement périnatal ? Cochez toutes les cases qui s'appliquent :

- [ ] ICPA - International Chiropractic Pediatric Association
- [ ] ICA - International Chiropractors Association - Council on Chiropractic Pediatrics
- [ ] MAQ - Mouvement allaitement Québec
- [ ] AQCPP - Association québécoise de chiropratique en périnatalité et pédiatrie
- [ ] Autre (veuillez préciser)

* 43. Quelle(s) techniques(s) de traitement utilisez-vous pour traiter les enfants de 0-6 mois?

- [ ] Haute vélocité, Faible amplitude
- [ ] Faible vélocité, Amplitude variable
- [ ] Assistée avec instruments (Activator, Artrostim ou autre)
- [ ] Speader Board pour la région cervicale
- [ ] Techniques crâniennes
- [ ] Techniques non-segmentaires
- [ ] Technique des Hautes cervicales
- [ ] Mobilisations des tissus mous
- [ ] Kinesio-Taping
- [ ] Thérapies musculaires
- [ ] Autre (veuillez préciser)

* 44. Quelle(s) techniques(s) de traitement utilisez-vous pour traiter les enfants de 7-23 mois?

- [ ] Haute vélocité, Faible amplitude
- [ ] Faible vélocité, Amplitude variable
- [ ] Assistée avec instruments (Activator, Artrostim ou autre)
- [ ] Speader Board pour la région cervicale
- [ ] Techniques crâniennes
- [ ] Techniques non-segmentaires
- [ ] Technique des Hautes cervicales
- [ ] Mobilisations des tissus mous
- [ ] Kinesio-Taping
- [ ] Thérapies musculaires
- [ ] Autre (veuillez préciser)

* 45. Quelle(s) techniques(s) de traitement utilisez-vous pour traiter les enfants de 2-5 ans?

- [ ] Haute vélocité, Faible amplitude
- [ ] Faible vélocité, Amplitude variable
- [ ] Assistée avec instruments (Activator, Artrostim ou autre)
- [ ] Techniques crâniennes
- [ ] Techniques non-segmentaires
- [ ] Autre (veuillez préciser)
- [ ] Technique des Hautes cervicales
- [ ] Mobilisations des tissus mous
- [ ] Kinesio-Taping
- [ ] Thérapies musculaires

* 46. Quelle(s) techniques(s) de traitement utilisez-vous pour traiter les enfants de 6-12 ans?

- [ ] Haute vélocité, Faible amplitude
- [ ] Faible vélocité, Amplitude variable
- [ ] Assistée avec instruments (Activator, Artrostim ou autre)
- [ ] Techniques crâniennes
- [ ] Techniques non-segmentaires
- [ ] Autre (veuillez préciser)
- [ ] Technique des Hautes cervicales
- [ ] Mobilisations des tissus mous
- [ ] Kinesio-Taping
- [ ] Thérapies musculaires

* 47. Quelle(s) techniques(s) de traitement utilisez-vous pour traiter les enfants de 13-17 ans?

- [ ] Haute vélocité, Faible amplitude
- [ ] Faible vélocité, Amplitude variable
- [ ] Assistée avec instruments (Activator, Artrostim, Pièce pour la tête ou autre)
- [ ] Techniques crâniennes
- [ ] Techniques non-segmentaires
- [ ] Autre (veuillez préciser)
- [ ] Technique des Hautes cervicales
- [ ] Mobilisations des tissus mous
- [ ] Kinesio-Taping
- [ ] Thérapies musculaires

10. Avenue futures dans la recherche chiropratique pédiatrique

* 48. Selon vous, **en ordre d'importance**, quels devraient être les axes de recherche future en chiropratique pédiatrique?

- [ ] Conditions musculosquelettiques sur la fonction et la douleur à la région cervicale
- [ ] Conditions musculosquelettiques sur la fonction et la douleur à la région thoracique
- [ ] Conditions musculosquelettiques sur la fonction et la douleur à la région lombaire
- [ ] Conditions cliniques impliquant les extrémités des membres supérieurs et inférieurs
- [ ] Affections viscéro-somatiques
- [ ] Recherches sur des thématiques de sports
- [ ] Posture
- [ ] Habitudes de vie
- [ ] Problématiques musculosquelettiques en allaitement
- [ ] Les facteurs déterminants parmi les conditions neuromusculosquelettiques affectant le développement neuromoteur normal

49. En lien avec la précédente, quel serait, selon vous, un autre axe de recherche future en chiropratique pédiatrique?

Nous vous remercions d'avoir participer à ce sondage.

Bonne journée!
